# Patient-centered care in the Middle East and North African region: a systematic literature review

**DOI:** 10.1186/s12913-023-09132-0

**Published:** 2023-02-09

**Authors:** Reeham Ahmed Alkhaibari, Jennifer Smith-Merry, Rowena Forsyth, Gianina Marie Raymundo

**Affiliations:** 1 Sydney School of Health Sciences, Faculty of Medicine and Health, The University of Sydney, Camperdown, NSW Australia; 2 Nursing Department, College of Applied Medical Sciences, Taif University, Taif, Saudi Arabia; 3 Centre for Disability Research and Policy, Sydney School of Health Sciences, Faculty of Medicine and Health, The University of Sydney, Camperdown, NSW Australia; 4 Cyberpsychology Research Group, Biomedical Informatics and Digital Health Theme, School of Medical Sciences, Faculty of Medicine and Health, The University of Sydney, Camperdown, NSW Australia

**Keywords:** Patient-centered care, Middle East, North Africa, Systematic review

## Abstract

**Background:**

The need for patient centered care (PCC) and its subsequent implementation has gained policy maker attention worldwide. Despite the evidence showing the benefits and the challenges associated with practicing PCC in western countries there has been no comprehensive review of the literature on PCC practice in the Middle East and North African (MENA) region, yet there is good reason to think that the practices of PCC in these regions would be different.

**Objectives:**

This paper summarizes the existing research on the practice of PCC in the MENA region and uses this analysis to consider the key elements of a PCC definition based on MENA cultural contexts.

**Methods:**

Five electronic databases were searched (EMBASE, Cochrane, Medline, CINAHL and Scopus) using the search terms: patient OR person OR client OR consumer AND centered OR centred AND care. The MENA countries included were Bahrain, Iran, Iraq, Jordan, Kuwait, Lebanon, Oman, Palestine, Israel, Qatar, Saudi Arabia, Syria, United Arab Emirates, Yemen, Algeria, Egypt, Libya, Morocco, Tunisia, Djibouti, Pakistan, Sudan, and Turkey. Identified papers were imported to Covidence where they were independently reviewed against the inclusion criteria by two authors. The following data were extracted for each paper: author, year, location (i.e., country), objectives, methodology, study population, and results as they related to patient centred care.

**Result:**

The electronic search identified 3582 potentially relevant studies. Fifty articles met the inclusion criteria. Across all papers five themes were identified: 1) patient centered care principles; 2) patient and physician perceptions of PCC; 3) facilitators of PCC; 4) implementation and impact of PCC; and 5) barriers to PCC.

**Conclusion:**

The preliminary findings suggest that the concept of PCC is practiced and supported to a limited extent in the MENA region, and that the implementation of PCC might be impacted by the cultural contexts of the region. Our review therefore highlights the importance of establishing patient-centered care definitions that clearly incorporate cultural practices in the MENA region. The elements and impact of culture in the MENA region should be investigated in future research.

## Background

### Patient centered care

Patient centered care (PCC) was introduced as a concept in the 1970s [1. ], however it did not gain popularity until the United States-based Institute of Medicine (IOM) declared it as one of six dimensions necessary to achieve quality of care alongside safety, timeliness, effectiveness, efficiency, and equity [2. ] Globally, however, there is no agreed definition of PCC in the literature [3. ]. For instance, the Institute for Patient and Family Centered Care (IPFCC) provides a broad definition, defining PCC as “the approach to planning, delivering, and evaluating healthcare that is based on a partnership between health care providers, patients, and families” [4. ] while on the other hand, Epstein & Street’s [5. ] definition focuses more on the interpersonal interactions of the healthcare encounter, describing it as an approach to treating patients with respect, recognising their preferences, engaging and involving them, and providing them with knowledge about their illness, care and treatment. Both definitions emphasise a need to establish a therapeutic relationship between health providers and patients in order to collaboratively achieve desired outcomes. Delaney [6. ] states that PCC aims to create collaborative rapport and takes a comprehensive approach to meeting and acknowledging patients' values, increasing their participation and including them in decision-making. Patient involvement in care is also seen to be encapsulated by the phrase “nothing about me, without me” [7. , 8. ]. As indicated by the name of the Institute for Patient and Family Centred Care, discussed above, another form of PCC is family centered care (FCC). The FCC model is based on the same fundamental aspects as PCC, but instead of focusing solely on the patient, it views patients and their family members as the care clients [9. , 10. ].

Evidence suggests that PCC can lead to better outcomes including increased patient and staff satisfaction, reduction in medical errors, enhanced employee recruitment, increased employee retention, improved health status and reduced unnecessary tests and referrals [11. –17. ]. The Picker Institute has established eight concepts of patient-centered care: prompt access to care; efficient treatment; quality care and adaptive transparency; patient and family involvement; comprehensible health care knowledge and support, mutual decision-making and respect for patient preferences; emotional support, empathy and respect, and awareness of physical and environmental needs [18. , 19. ]. These concepts consider PCC at all stages of the patient's journey and their care environment.

The shift to PCC was part of an approach to health care based on patients’ rights and aligned with the World Health Organisation (WHO) International Declarations of Geneva and Alma Ata [20. , 21. ]. The rationale for adopting this approach was the global shift in health patterns from infectious diseases to chronic diseases, which increased demand for health care, and fears that the healthcare system might become overwhelmed when meeting these demands [22. ]. A PCC approach views patients as the core of the healthcare system and part of the solution to improving the quality of care and, therefore, its effectiveness [23. , 24. ]. An adoption of PCC, it was argued, would prioritise patients’ rights in decision making, make care more targeted and motivate patients to take control and become experts in managing their conditions [25. ]. Collaboration and relationship building between health providers and patients is key to the adoption of PCC. The elements of this collaborative relationship are explained in Carman's [24. ] 'Framework for Patient and Family Engagement in Health and Health Care'. This framework points to a relationship where health providers’ and patients’ values, experiences, and perspectives in disease prevention, diagnosis and treatment are combined and enacted through providing clear information, communication, establishing goals, and participating in decision making, to proactively manage health. This relationship builds open communication to ensure that patients understand the risks and benefits associated with their health choices [24. ].

### The health care system and PCC in the Middle East and North Africa region

According to Webair [26. ] middle eastern countries have had a long history of practicing the principles behind PCC as the provision of health care has been influenced by the principles of Islam since the medieval period. For instance, medieval Islamic medical practice involved treating patients without discrimination related to gender and identified ethics as a prerequisite for health provider practice. This resulted in high quality care provision, however in the present time, the PCC performance of middle eastern countries has been considered poor compared to other western countries [26. ]. This poor performance may result from a wide range of factors, including changes to disease patterns, population growth and the increased demand on health services, cultural factors, lack of financial resources, administrative and organisational reasons, the inaccessibility of medical treatments and healthcare services due to poverty, and in some countries, instability from war [27. –29. ].

In 2000, the World Health Organization (WHO) attempted to rank the health system performance of 191 countries based on five indicators including population health, health inequality, health system responsiveness to population needs, distribution of this responsiveness, and fairness in financing [30. ]. Many Middle East and North Africa (MENA) countries were among the top 30 best performing health systems including, Oman, Saudi Arabia, the United Arab Emirates, and Morocco but others were poorly rated including Pakistan, Sudan, and Djibouti [30. ]. Most countries in the MENA region have a split healthcare system with public and private funding of service delivery [27. ] and comply with the Alma- Ata Declaration of 1978 to provide ‘Health for All’ [28. , 31. ]. However, there is disparity in the quality of healthcare among these countries especially in rates of government funding [27. ]. For example, oil rich countries such as Saudi Arabia [32. ] can allocate oil revenue to improve health care, provide health care programs [28. , 33. ], and universal access and affordable care [28. ]. These all improve patients’ access to and utilisation of the health system and therefore improve the quality of care [28. ]. On the other hand, in low-income countries in the MENA region, such as Pakistan [34. ], the health system remains in a fragile state due to factors which include lack of resources, poor structural management, lack of equity, gender insensitivity, and inaccessible and unaffordable health services [35. ]. Moreover, currently, four countries in the MENA region—Syria, Iraq, Libya, and Yemen—are in a state of war[36. ]. War and conflict affect health system quality [37. ] due to the destruction of health infrastructure, a shortage of health care providers, and lack of medical equipment and medicines [38. –40. ]. These factors can have varying degrees of impact on genders and communities [29. ]. Therefore, while health systems in the MENA region need to adopt PCC methods to improve care delivery and governance by informing patient and provider education and policies [41. ], they are not starting from an even base, and PCC needs to be implemented with an understanding of broader system development and characteristics. Webair [26. ] also points out that the current published literature in the MENA region applies a western definition of PCC with the absence of a definition of PCC reflecting the culture of the region which includes a stronger emphasis on family and religion. These barriers are key features that must be considered when understanding how to improve the implementation of PCC in MENA countries.

This literature shows that there are possible structural and cultural limitations on the adoption of PCC in the MENA region but we lack a clear understanding of the factors impacting PCC implementation. There is also a necessity for establishing a definition of PCC based on the culture of the region which incorporates the particular circumstances impacting the implementation of PCC. The purpose of this review is to start to address these limitations by documenting the current published literature on PCC in the MENA region and to use this analysis to consider the essential elements of a MENA focused definition of PCC.

## Method

A systematic literature review was performed of the published literature on patient centred care in the Middle East and North Africa. Systematic review described by Petticrew and Roberts [42. ], p., 15) as “a method of critically appraising, summarising, and attempting to reconcile the evidence”. This method is beneficial because it seeks to summarize published research that has been done in a certain field of study, provide an overview of the research field, highlight areas where research has been conducted, and identify knowledge gaps. This approach also allows us to take from this literature the key elements of PCC as practiced in the MENA region. For the purposes of this research, we define PCC in relation to the eight principles of PCC articulated by the Picker Institute, discussed earlier [19. , 43. ]. We followed the scoping review process suggested by Peters et al. [44. ] to conduct this review.

RA is an academic researcher who has spent much of her life living and working in Saudi Arabia. JSM, RF and GR are academic researchers with experience of Australia and other international health systems research contexts.

### Literature search

A search was carried out in relevant electronic databases (EMBASE, Cochrane, Medline, CINAHL and Scopus) for studies that focus on patient-centered care in the MENA region and were published up to January 2021. The following terms were developed with the assistance of the university librarian to identify publications associated with patient-centred care: patient OR person OR client OR consumer AND centered OR centred AND care. The MENA countries of Bahrain, Iran, Iraq, Jordan, Kuwait, Lebanon, Oman, Palestine, Israel, Qatar, Saudi Arabia, Syria, United Arab Emirates, Yemen, Algeria, Egypt, Libya, Morocco, Tunisia, Djibouti, Pakistan, Sudan, and Turkey were used as keywords combined with the terms above in order to limit findings to the MENA region. Iran and Turkey were included in the study as they are sometimes included in the definition of the MENA region and share cultural and religious characteristics in common with the other countries [45. –47. ]. Results imported from the databases were stored in the reference manager software EndNote and then imported to the systematic review software Covidence where duplicates were removed, and remaining papers screened for inclusion.

### Study selection

The inclusion criteria were: 1) studies focusing on patient-centered care; 2) studies published in English and/or Arabic (the two languages spoken by the research team); 3) studies published in peer-reviewed journals; 4) studies that sampled participants (including patients, family members, health providers, and nursing and medical students 5) any study design that utilized qualitative, quantitative, or mixed methods approaches to data collection; and 6) studies published in the MENA counties. In addition, studies reported family centered care were also included in the review. Two reviewers (RA and GR) independently scanned the titles and abstracts to assess the relevance of the studies in relation to the inclusion criteria. Studies that potentially met the inclusion criteria were retrieved for full text screening. Any disagreement between reviewers was resolved by discussion between the reviewers, bringing in a third person (JSM) where necessary. Studies were excluded if they did not focus on PCC, were not conducted in the MENA region, included no data from participants, were conducted in languages other than Arabic or English, where full articles were not accessible, or were other types of papers (including reviews, systematic reviews, commentary, editorials, protocols, opinion pieces and conference papers).

### Data extraction

The data were extracted and recorded into a spreadsheet in MS Excel from the Covidence software. Collected data recorded were the author, year, location (i.e., country), objectives, methodology, the study population, and findings of each study. The main themes across the papers were identified through an open coding scheme, with five categories created based on these key themes: patient-centered care principles; patient and physician perceptions of PCC; facilitators of PCC; implementation and impact of PCC; and barriers to PCC.

## Results

### Description of the literature

**Fig. 1:**
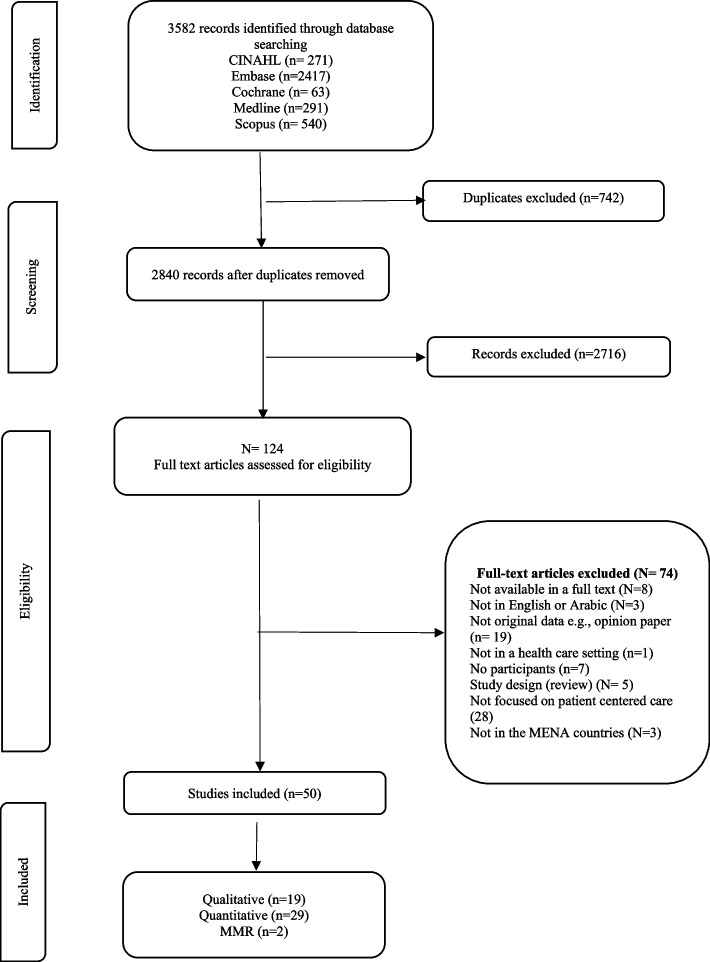
Flow PRISMA diagram for search and selection process

The database search initially resulted in the identification of 3582 articles (Fig. 1). Fifty articles met the inclusion criteria and were included in the review. Of the 50 articles, 29 used quantitative methods, 2 used mixed methods and 19 used qualitative methods. Studies were conducted in Iran (13), Israel (7), Saudi Arabia (6), Jordan (5), Pakistan (4), United Arab Emirates (2), Kuwait (1), Oman (1), Qatar (1), Turkey (1), Egypt (1) and Palestine (1). The seven remaining studies reported comparative data from multiple countries in the Middle East. Data were generated using a wide range of methodologies from qualitative interview designs, qualitative focus group designs, mixed qualitative methods studies, cross sectional surveys, quasi-experimental, randomized designs, non-experimental design, and mixed designs. Details of these studies are provided in Table 1. The sample sizes used ranged from 9 to 829 participants, with the smaller samples from qualitative studies and the largest sample from a multisite database study. The sample populations included health providers (20), patients and health providers (11), patients and family members (14), medical and nursing students (4), clinical and non-clinical staff (1), and academic and clinical experts (1).

**Table 1:** Summary of studies included in the review updated

Author and year	Country	Aim	Participant type	Study design and methods
Abdelhadi and Drach-Zahavy, 2012 [48. ]	Israel	To test a model that suggests that the ward’s climate of service facilitates nurses’ patient‐centred care behaviours through its effect on nurse work engagement	Nurses	Cross-sectional multi-level (nurses within wards) design
Abdel-Tawab and Roter, 2002 [49. ]	Egypt	To examine the feasibility, acceptability, and effectiveness of client centered models of communication in 31 family planning clinics in Egypt	Physicians and clients requesting family planning methods	Quantitative interviews and questionnaire
Abdulhadi et al., 2007 [50. ]	Oman	To explore the perceptions of type 2 diabetes patients regarding medical encounters and quality of interactions with their primary health-care providers	Diabetic patients	Qualitative: Explorative study using Focus group
Ahmad et al.,2015 [51. ]	Pakistan	To assess the leaning of medical students towards either a doctor-centred or a patient-centred care, and to explore the effects of personal attributes on care such as gender, academic year, etc	Medical student	Cross-sectional
Akkafi et al., 2019 [52. ]	Iran	This study measured the attitudes of patients’ family and personal caregivers (FPCs) and psychiatrists toward patient-centred care	Patients’ FPCs and psychiatrists	Descriptive questionnaire-based study
Akturan et al., 2016 [53. ]	Turkey	To investigate the effect of the BATHE therapeutic interview technique on the empowerment of diabetes mellitus patients in primary care	Diabetic patients	A cluster randomised controlled study
Alabdulaziz and Cruz, 2020 [54. ]	Saudi Arabia	To explore the perceptions of nursing students toward family-centered care in Saudi Arabia	Undergraduate nursing students	Cross-sectional survey method
Alabdulaziz et al., 2017 [55. ]	Saudi Arabia	To explore family-centred care in the Saudi context from the perspectives of paediatric nurses	Paediatric nurses	Mixed method sequential exploratory design
Alameddine et al., 2020 [56. ]	Dubai	To explore the perceptions of physicians in regard to SDM in a large private hospital network in Dubai, United Arab Emirates	Health care providers	Cross sectional
Albougami et al., 2019 [57. ]	Saudi Arabia	To examine the perceptions of expatriate nurses in Saudi Arabia regarding the relationship between cultural competence and patient-centred care	Expatriate nurses	Cross-sectional descriptive correlational survey
Alhalal et al., 2020 [58. ]	Saudi Arabia	To assess the predictors of patient‐centred care provision among nurses working in an acute care setting	Nurses	A cross‐sectional predictive design
AlHaqwi et al., 2016 [59. ]	Saudi Arabia	To determine the perceptions of patients on whether they receive sufficient information about their medical problems, their preferences to obtain information, and factors that may influence their preferences	Patients attending the primary health center during the study	Cross-sectional, questionnaire-based study
Aljaffary et al., 2020 [60. ]	Saudi Arabia	To measure patient perceptions of shared decision-making practices during clinical encounters in Saudi Arabia	Patients and physician	Cross sectional
Al-Momani, 2010 [61. ]	Jordan	To assess the satisfaction of mothers with paediatric health care at the Paediatric Unit (PU) of Paediatric and Maternity Hospital, Al- Mafraq, Jordan	Mothers at a Paediatric Unit	A descriptive/ correlation survey
Alshahrani et al., 2018 [62. ]	Saudi Arabia and Australia	To explore the nature of relatives’ involvement in the care of patients in acute medical settings in Australia and Saudi Arabia and to explore the perceptions, attitudes, and experiences of nurses	Female patients, their relatives, and nurses	Qualitative: Ethnography (Observation and interview)
Aminaie et al., 2019 [63. ]	Iran	To explore the perception of barriers to decision making in Iranian patients with cancer regarding their care	Cancer patients	Qualitative: Incorporated individualized in depth and semi structured interview
Baig et al., 2020 [64. ]	Pakistan	To explore significant facilitators and barriers to effective SDM as perceived by endocrinologists	Endocrinologists	Qualitative: In depth interview
Bentwich et al., 2018 [65. ]	Israel	To explore whether normalization coping mechanisms exist among formal caregivers, reveal differences in its application among cross-cultural caregivers, and examine how coping mechanisms may related to implementing PCC for people with disability	Caregivers consisted of nurses, nurses’ aides, occupational therapists, and physiotherapists	Qualitative
Bentwich et al., 2019 [66. ]	Israel	To explore caregivers’ verbal patterns for potential links between respect for the personhood of patients with dementia and caregiver use of figurative language	Caregivers three groups: Arabs, immigrants from the former Soviet Union and Jews born in Israel	Qualitative: using a discourse analysis of semi-structured interviews
Cheraghi et al., 2017 [67. ]	Iran	To explore the process of providing patient-centred care in critical care units	Nurses, patients, and physician	Qualitative: used a grounded theory method semi structured interview
Dormohammadi et al., 2010 [68. ]	Iran	To identify the most important expectations that patients have about their physicians	Hospitalized and ambulatory patients	Quantitative
Drach-Zahavy, 2009 [69. ]	Israel	To assess the moderating effect of care orientation on the relationship of PCC to nurses’ physical and mental health	Registered Nurses	Cross sectional study
Esmaeili et al., 2014 [70. , 71. ]	Iran	To explore nurses’ attitudes and experiences towards the barriers to achieving patient centred care in the critical care setting	Nurses working in intensive care units	Qualitative: In depth semi-structured interview
Esmaeili et al., 2014 [70. , 71. ]	Iran	To explore the perception of nurses working in critical care units about patient-centred care, which is a crucial factor in attaining quality in nursing care	Nurses in critical care	A qualitative exploratory study using semi structured interview
Esmaeili et al., 2016 [72. ]	Iran	To explore cardiac patients' perceptions of patient‐centred care	Cardiac patients	Qualitative: Semi structured interview
Feeg et al., 2016 [73. ]	United States, Australia, and Turkey	To describe and compare how healthcare providers from three countries with varied cultural and healthcare systems perceive the concept of FCC by measuring attitudes, and to develop a psychometrically sound measure that would reflect “family-centredness.”	Providers working in paediatrics	multi-site comparative nonexperimental design
Ghaffari et al., 2020 [74. ]	Iran	To determine the predictive values of patient‐centred communication and patient characteristics on body image perception in postmastectomy patients	Surgically treated breast cancer patients admitted to the oncology departments of two hospitals in Iran	Predictive correlational study
Ghiyasvandian et al., 2018 [75. ]	Iran	To explore therapeutic communication between patients and nursing students in the Iranian context through the perception of nursing students, nursing instructors, and patients	Nursing students, nursing instructor, and patients	Qualitative: In depth semi structured interview
Hayajneh et al., 2014 [76. ]	Jordan	To describe the impact on professional caregiver burden of adopting person‐centred care approaches for people with Alzheimer's disease	Professional caregivers	A qualitative descriptive phenomenological approach using semi‐structured interviews
Hijazi et al., 2018 [77. ]	Jordan	To examine the impact of applying quality management practices on patient centeredness within the context of health care accreditation and to explore the differences in the views of various health care workers regarding attributes affecting patient-centred care	Clinical/nonclinical hospital staff	A multiple-case study design
Joolaee et al., 2010 [78. ]	Iran	To describe how Iranian patients and their companions explain their lived experiences with caring relationships in a central teaching hospital in Tehran, Iran	Patients and their companions	phenomenological approach was used and semi-structured interviews
Khullar and Coughlan, 2018 [79. ]	Kuwait	To understand the types of narratives and treatment approaches that may contribute to inadequate service delivery	Individuals providing mental health services	Qualitative: using open-ended, semi-structured interviews
Lipovetski & Cojocaru, 2020 [80. ]	Israel	To shed the light on the integration of caregiver- patient perspectives regarding decision-making processes, and the barriers and facilitators to their implementation in chronic-care practices in Israel	Patients with colorectal cancer and Oncologists	Mixed method Explanatory sequential design
Mahboub et al.,2018 [81. ]	Dubai	To review current patient-centred practices for outpatients in both private clinics and public hospitals in Dubai	Patients had appointments in various outpatient departments (OPD) including medical, surgical, and general	Independent survey consisting of self-administered questionnaires
Manchaiah et al., 2014 [82. ]	Portugal, India, and Iran	To examine and compare audiologists’ preferences for patient-centredness in Portugal, India, and Iran	Audiologists	Cross sectional
Medina-Artom and Adashi, 2020 [83. ]	Israel	To compare provider and patient perceptions of the extent to which care in Israeli IVF units is patient centred	Providers and patients	Quantitative using questionnaire
Mohamed et al., 2013 [84. ]	Qatar	To assess the effectiveness of a culturally sensitive, structured education programme (CSSEP) on biomedical knowledge, attitudes, and practice measures among Arabs with type two diabetes	Patients with type II diabetes	A randomized controlled trial
Mole et al., 2016 [85. ]	UK, Egypt, and India	To characterise understandings of ‘good communication’ in future doctors from medical schools in three contextually contrasting continents and test the hypothesis that there would be a lack of global consensus on what constitutes ‘good communication’	Students in the clinical years of training (Years 3–6) who had personal experience with doctor–patient interactions	Qualitative: A mixed method using first focus groups and then interviews
Obeidat and Lally, 2018 [86. ]	Jordan	To determine Jordanian physicians' perceived barriers and facilitators to patient participation in treatment decision-making	Physician	Quantitative: A cross sectional exploratory survey design
Rahman et al., 2019 [87. ]	Pakistan	To examine the perspectives of 18 health care providers (nurses, consultant doctors, residents, radiologists, and physiotherapists) and 18 patients regarding best practices for patient‐centred care in a free private hospital in Pakistan, to understand congruence between provider and patient perspectives	Patients and providers	Qualitative: Focused group interview
Rasha et al., 2009 [88. ]	Saudi Arabia and US	To use the Communication, Curriculum, and Culture Survey (C3) to perform a pilot cross-cultural comparison of the patient-centredness of the hidden curriculum between a Saudi medical school and 9 U.S. medical schools	Senior Saudi medical students in their (6^th^) last year	Quantitative
Rassouli et al., 2020 [89. ]	Iran	To examine nurses' perceptions of the components of patient-centred care and its delivery	Nurses	Qualitative descriptive research using face to face semi structured and filed note interview
Rehman eta l., 2017 [90. ]	Pakistan	To determine health care professionals’ views on patient centricity, quality improvement and advancement required for life satisfaction in the health care sector	HCPs working in tertiary care hospital and after non solo clinic	Descriptive cross sectional
Sadati et al., 2016 [91. ]	Iran	To explore the nature of doctor-patient interaction (DPI) in one educational hospital in Iran, according to the views of patients and their relative	Patients and their relatives	Qualitative: using the Carspecken’s critical ethnography
Schattner et al., 2006 [92. ]	Israel	To determine patient priorities in medical care	Hospitalized and ambulatory patients	Quantitative
Sultan et al., 2018 [93. ]	Palestine	To investigates the provision of PCC among hospital doctors in the developing and politically unstable country of Palestine	Doctors	Descriptive, cross-sectional research
Topaz et al., 2020 [94. ]	Israel and US	To understand the extent to which current health information technology (HIT) systems are supportive of PCC and how PCC should be supported by HIT in the future	Academic and clinical experts (Physician, Nurse, Human factors analysis/human computer interaction experts. Other (socio-technical domain & social work)	A qualitative descriptive method interview
Yasein et al., 2017 [95. ]	Jordan	To evaluate patient-centredness and communication skills from the patients’ point of view and that of the physicians’ point of view and compares the two outcomes	Patients and residents	Quantitative: A cross sectional study
Zisman-Ilani et al., 2020 [96. ]	Israel, Jordan, and US	To understand the beliefs, perceptions, and practices related to shared decision making (SDM) and patient-centred care (PCC) of physicians in Israel, Jordan, and the United States	Physicians	Quantitative: using a web-based survey
Zolfaghari et al., 2015 [97. ]	Iran	To study the effect of two educational methods (family-centred and patient-centred) on some complications that occur during haemodialysis	Patients	Clinical trial of quasi-experimental type

### Patient centered care principles

The key elements of PCC identified in the studies were: treating patients as an individual [67. , 70. , 89. ] having empathy towards their conditions, advocating on their behalf and providing care with flexibility to meet patients’ needs, incorporating patients’ expectations and preferences [70. , 72. , 78. , 89. ]. Papers also highlighted attentiveness to patient needs, including physical, religious, and social needs [70. , 72. , 89. ]. Other key areas were health professionals’ competency and expertise in protecting patients from harm, reducing hospitalization and costs of care [67. , 70. , 72. ], complying with patients’ rights [70. , 72. ], effective communication [67. , 72. , 78. , 89. ], and empowerment of patients through providing education [67. ].

### Patient and health provider perceptions of PCC

Several studies showed that patients and their relatives had a positive attitude toward patient involvement in care [52. , 59. , 62. , 80. ]. Schattner et al. [92. ] added that patients like to be informed about their health and participate in shared discussions and decision making about the care they receive. Dormohammadi, Asghari & Rashidian’s [68. ] study of hospitalized and ambulatory patients in Iran noted that patients prioritized health professional competence and positive attitudes. However, in the study by Joolaee et al. [78. ], which focused on Iranian patients and their companions' experiences with caring relationships with health professionals, patients prioritized health professional positive behaviour and emotional support over competence.

There were variations regarding patients’ and health providers’ perspectives on the way in which patient-centered care was practiced. For instance, Yasein et al. [95. ] wrote about patient and physician perspectives on patient-centeredness and communication skills in Jordan. The study found that patients rated many aspects of patient-centeredness and communication skills lower than junior doctors. Another study conducted in Israeli IVF units found that there were differences between the perceptions of patients and providers regarding the provision of the different aspects of PCC and the extent to which each dimension of patient-centred care was applied. Dimensions assessed were for example, emotional support, respect for patient values and needs and provision of information and explanation of the 10 dimensions introduced by Picker Institute. Patient scores were lower than those of providers except for continuity of treatment and professional competence [83. ]. This variation was also noticed in the practice of FCC. Several studies explored health provider perceptions of FCC. Cross-national differences in the perception of FCC were identified among healthcare providers in the United States, Australia, Turkey, and Saudi Arabia[54. , 73. ]. A study by Alabdulaziz et al. [55. ] investigated FCC in Saudi Arabia from the paediatric nurse perspective which showed that nurses acknowledge the significance of family-centered care elements however they find it difficult to apply this model in their daily practice. Overall, however, this variation in attitudes to PCC between patients and practitioners and across studies shows that there is no clear understanding of PCC common within the practices reported in the existing literature and, in relation to the Picker Institute definition, we can see that PCC is not fully implemented in the MENA region.

### Facilitators of PCC

Factors influencing the practice of PCC were related to patient gender [59. , 60. ] and the broader sociodemographic characteristics of physicians [49. , 51. , 58. , 93. ], patient support networks [80. , 86. , 87. ], patient-provider communication [64. –66. , 75. , 76. , 85. ] and practitioner work environment [48. , 69. , 71. , 98. ].

Two studies showed that patient gender was significantly associated with their preference to be informed of treatment plans and participate in decision making where women were more likely to be involved than men [59. , 60. ]. However, this was not a universal finding, with Lipotevski and Cojocaru’s [80. ] study of patients with colorectal cancer and oncologists in Israel showing no association between preferences towards PCC and gender. This shows that the impact of gender on PCC may reflect the practice environment or prevailing social context of the country in which PCC is being implemented.

Practitioner demographic, education and practice context impacted on PCC uptake. Ahmad et al. [51. ] investigated the relationship between demographic characteristics and the attitude of undergraduate medical students in the pre-clinical years and in clinical years towards PCC in Pakistan and found that foreign students studying in private medical schools were more in favour of PCC compared to local students. Physician age was also a factor, with Abdel-Tawab and Roter’s [49. ] study of a family planning program in Egypt suggesting that young physicians prefer to work in a patient-centered way. However, Alhalal et al. [58. ] found that older providers were better at showing empathy, communicating effectively, responding to patients’ needs, and sharing decisions with patients. Sultan et al. [93. ] suggest that a broad range of demographic and educational factors influence physician views of the importance of PCC components. Relevant factors were practitioners’ job title, age, marital status, and private hospital context, along with familiarity with PCC.

Several factors influenced health providers decisions to practice PCC. This included the availability of evidence, health professionals’ values, environment or context-related factors, such as cultural beliefs [56. ]. Involving clinical and non-clinical staff in service accreditation processes was also a significant factor in delivering PCC in health organization settings [77. ].

For patients, PCC was facilitated by receiving emotional support from family and others [80. ], family involvement in care more generally [87. ] and patients bringing someone with them during a consultation [86. ]. Several studies investigated patients' experience of PCC within health services in different settings, including family planning clinics [49. ], private clinics, public hospitals [81. ], and paediatric units [61. ]. Factors contributing to patient satisfaction were positive talk [49. ], timely services and appointments, and provision of clear explanations regarding the patients’ medical conditions [81. ].

With regard to facilitating factors associated with the work environment, Esmaeili et al.’s [70. ] study of nurses working in critical care in Iran noted that nurses reported that organizational recognition of staff shortages, providing guidelines, support to staff and the presence of PCC role models in the workplace were facilitators of PCC. One study investigated the perceptions of expatriate nurses in Saudi Arabia concerning the relationship between cultural competence and patient-centered care. It found that there was a positive association between cultural competency and providing individualized care [98. ]. Abdelhadi and Drach-Zahavy [48. ] suggest in their study of nurses in north Israel that establishing a better climate of service and work engagement would influence PCC provision by providing support, training, and incentives. Alhalal, Alrashidi & Alanazi [58. ] suggest that high levels of structural empowerment and compassion, satisfaction and low burnout are important factors that influence the provision of PCC by staff. In another study by Drach-Zahavy [69. ], nurse mental health improved when providing a high quality care and high PCC and declined when the opposite was true.

In addition to doctors’ attitudes, the papers emphasised that effective communication skills, including empathic and therapeutic communication, are essential elements of successful PCC by health care providers [64. ]. Two studies explored PCC in relation to the implementation of coping strategies and supportive language by cross cultural care givers of people with dementia. Results showed that Arab care givers used figurative language and coping strategies embedded in PCC approaches which viewed people with dementia as individuals with unique needs [65. , 66. ]. Hayajneh et al. [76. ] found that empathetic relationships by staff with patients with Alzheimer’s overrides stresses and allows implementation of effective coping strategies which reduce the burden of caring for people with Alzheimer’s. Ghiyasvandian et al. [75. ] found that therapeutic communication assists health providers to provide PCC that aligns with patient needs. It establishes trust with patients through asking permission before providing care, guides conversations to patient needs, is responsive to their needs, and provides personalised communication influenced by religious, cultural, and professional values. Utilizing patient-centered communication strategies was significantly associated with better health outcomes [74. ]. Rahman et al. [87. ] found several key physician behaviours that facilitate PCC including positive attitudes, allocating time for consultation, cultural responsiveness and the use of simple language and props to demonstrate procedures. However, a study conducted to compare perceptions of 'good communication' across medical schools in different sociocultural contexts found there was variation in what is considered to be good communication [85. ]. This variation was associated with different views on the role of family, gender, and emotional expression across the study sites. One study compared PCC in the hidden curriculum in a Saudi medical college with nine US medical colleges. The results suggested that the hidden curriculum in Saudi schools is more physician than patient-centered [88. ].

### Implementation and impact of PCC

The impact of different PCC strategies on patients and physicians was a significant theme in the identified papers, however because of the disparate nature of the topics and settings, the papers approached this theme from a variety of angles. Two studies utilised the BATHE interview technique (Background, Affect, Troubling, Handling, and Empathy) and empowerment educational sessions to empower diabetic patients [53. , 84. ]. The studies used HbA1c, BMI and patient empowerment scores as outcomes and found mixed results, with one finding a reduction in HbA1c and BMI values as a result of the intervention (− 0.55 mmol/L, *P* < 0.0001; − 1.70 kg, *P* = 0.001) [84. ] and another finding no significant differences [53. ]. However, other improvements were found such as knowledge of diabetes, attitude towards diabetes, and in practice such as patient compliance with the treatment [84. ] and empowering diabetic patients [53. ]. Another study based in Iran assessed the effect of FCC and PCC on complications during haemodialysis [97. ]. Findings suggested that family-centred education led to improved treatment outcomes including reducing haemodialysis complications and depression and increasing satisfaction and self-care.

### Barriers to PCC

Papers reported barriers to PCC as arising from 1) the individual patient and their support environment, 2) the health professional and 3) the organizational environment. Individual patient barriers included patients’ low levels of education, low health literacy and lack of motivation to take an active role in their health [50. , 64. , 80. ]. Low literacy was identified as a barrier to managing health care by both patients and health care providers. Abdulhadi et al. [50. ] argued, in an Oman-based study, that patients reported that they could not interact with health care providers due to their low levels of education. They found that patients with low levels of education hesitated to interact with their health care providers as they feared that their interference would negatively affect their relationship with the healthcare provider [50. ]. Two other studies cited lack of knowledge or low health literacy and self-efficacy as barriers to PCC [80. , 96. ]. However, another study reported physicians’ perceptions that there was no relationship between patient level of education and participation in decision making [64. ].

Papers reported that patient centred care is related to patient beliefs about their illnesses or the nature of health care interactions. For example, patients do not acknowledge the seriousness of their illnesses or have expectations of healthcare encounters which focus on treatment rather than consultation and this affects the patient’s interactions with the health care professional [80. , 86. , 96. ]. Two studies noted patients’ understanding of the health care relationship as a barrier to PCC. This resulted from medical dominance and patient views of health care provider authority [63. , 80. ]. Medical dominance was defined by Iranian cancer patients as the provision of inadequate information to patients, perceived authoritarian behaviours in physicians, as well viewing patients as objects for financial gain [63. ].These factors resulted in the inability to discuss physician decisions [63. ]. For patients this led to fear and despair about the lack of alternative treatment options.

A number of papers reported the impact of culture on patient centered care. Aminaie et al. [63. ] noted barriers to PCC that were associated with patient ethnicity, faith, and language. Their Iranian study explored how cancer patients perceive barriers to participation in decision-making and found that patients preferred to receive care in a familiar health care organization so they would feel comfortable in communicating with their health care professional to seek more information. Key to this was a concern that the health care professional would not dismiss their faith [63. ]. Meanwhile, in a study by Alabdulaziz et al. [55. ], nurses working in Saudi Arabia with different languages, religions and cultures to their patients, was viewed as a barrier to involving patients and their families in decision-making. Baig et al. [64. ] reported that health providers hold respected positions in Pakistani culture and were encouraged to make decisions without involving patients.

The role of family members and caretakers as a potential barrier to PCC in health care interactions was reported in several studies. One study investigating Jordanian physicians' views of the barriers and facilitators to patient involvement in decision making found that 65.5% of physicians reported involving the family in decision making as a barrier to PCC as families would interfere and make decision on behalf of the patient [86. ]. Baig et al. [64. ] noted that family involvement in Pakistan is two-edged as involving family can help in managing patients’ conditions however it can also be counterproductive if handled incorrectly.

Many studies reported health care professional behaviours as a barrier to PCC [63. , 80. ]. These included dismissing patient and family concerns, negative attitudes toward PCC, a lack of eye contact when interacting with patients, poor sharing of information and delays in care provision [50. , 63. , 80. ]. Patients also noted a lack of privacy during interactions with the health care provider, poor communication as the health professional was busy taking notes, poor encouragement of patient involvement, and poor provision of information or explanation of patient conditions [50. , 63. , 64. ].

A lack of professional motivation on the part of health practitioners has also been described as a barrier impacting the implementation of PCC. This was associated with their lack of interest in the profession, low salaries, staff shortages, not receiving financial incentives, workload burden and dissatisfaction [64. , 71. ]. A lack of collaboration between the healthcare team members [71. , 87. ] as a result of poor communication between physicians and nurses due to gender sensitivity was also reported as a barrier to PCC [87. ].

Limited understanding of the concept of PCC was cited as a barrier to the implementation of PCC in two studies [80. , 96. ]. Zisman-IIani et al. [96. ] found that only 40% of Jordanian physicians and 71% of Israeli physicians understood the PCC concept. Studies also commented on healthcare provider lack of expertise and competence regarding patient conditions [50. ]. Abdulhadi et al. [50. ] noted that patient perceptions of health care providers’ lack of knowledge of diabetes was due to the short consultation time, infrequent physical examinations, and a misguided belief that diabetes is not a serious health condition.

Various organizational barriers towards PCC were reported across studies. The lack of time resulting from high workloads and patient loads led to practitioners being less able to share information with patients [55. , 64. , 71. , 80. , 86. ]. Other factors which inhibited PCC included a lack of organisational and managerial support, scarce resources and facilities, administrative blockages, lack of staff, and limited time to establish rapport and negotiate care [55. , 63. ]. Two studies described long waiting times as a barrier to PCC [50. , 87. ] which was as a result of bed shortages, high patient loads, lack of free services and a lack of referral practices in the health care system [87. ]. A lack of training and guidelines, and resources to support PCC was also a significant organizational barrier [55. , 71. , 80. , 96. ].

## Discussion

This review systematically assessed 50 articles which had a significant focus on patient-centered care practice in the MENA region. The included studies focused primarily on patient-centered care principles; patient and physician perceptions of PCC; facilitators of PCC; implementation and impact of PCC; and barriers to PCC. Numerous barriers and facilitators were reported across the studies. While facilitators related to all groups, facilitators were most commonly reported to be related to physician attitudes and practice, there is an ongoing need for literature that explores the facilitators of PCC related to patients and organizations, in order to offer a more multi-layered approach to improving PCC.

### The cultural context of MENA and PCC

Our findings suggest that culture plays a significant role in influencing patient provider relationships and therefore the extent to which PCC is practiced. Patient-provider interactions are rooted within prevailing cultural and religious norms and influenced by time and setting [99. , 100. ]. This profoundly influences patient attitudes, beliefs, and health-related practices [101. ]. Social behaviour and practices in much of the MENA region are governed by cultural norms and religion [64. ] whereby community, family, and religion play a significant role in decision-making [87. , 102. ]. Following the dimensions of culture introduced by Hofstede, Hofstede, and Minkov ([103. ], p., 103–104), Middle Eastern countries are characterised by dependent collectivism where decisions related to health are made collectively by family members [50. , 104. ]. With respect to the dimension of power distance, defined as the degree to which less powerful individuals expect and accept an unequal power distribution ([103. ], p., 61), this was evident in a country like Pakistan where health providers, such as doctors and elderly family members, held an authoritative position. Consequently, in such societies doctors are viewed as the “instruments of God” and “paternal figures” and therefore have the power to make decisions, which impacts on the practice of PCC [64. , 86. , 100. ]. Accordingly, family systems and the position of authority that a doctor holds forms the basis for medical decision making in many MENA countries [105. ].

We also observed in our review that family involvement was viewed as both a barrier and a facilitator to PCC by both patients and health providers [62. , 64. , 86. ]. Family members in countries in the middle east play a protective role for their hospitalised members by providing spiritual and financial support. On the other hand, a family might interfere with patient treatment plans [50. , 86. ]. Consequently, family members may be the ones to make the decisions for the patient or patients may delegate their family to participate in the decision-making process [64. , 86. , 104. , 105. ]. The role of family in understanding PCC in the MENA region is therefore essential to a developing contextual definition of PCC.

Islam is the most prevalent religion in the MENA region [106. ]. In this religion, the importance of hygiene, diet, and exercise is taught, and people are encouraged to practice healthy behaviours. However, the provision of health care might also be hindered by certain beliefs, including the patient preferences of the provider’s gender [107. ] and gender may therefore play a key role in patient-provider interactions which impacts on the practice of PCC [108. ]. In societies where gender separation exists it is common for women to choose a female provider over a male provider and involve a male family member in decision making [104. , 107. , 109. , 110. ]. Failing to provide health care services in accordance with gender-based, religious and cultural needs is therefore considered a barrier to care and will impact on PCC. This finding is supported by several studies conducted with Muslim women in western countries, such as the United States and Australia, which have shown that women seek care if it is aligned with their religious and cultural values [101. , 111. –113. ]. These findings show that gender is a key part of the cultural operationalisation of PCC in the MENA region and must be considered within the practice of PCC.

### PCC practice facilitators and barriers

The key facilitators of PCC found in this review were 1) the establishment of a therapeutic relationship between the health provider and patient 2) practices which treated the patient as a whole person, 3) respect, including of patient preferences and values, 4) effective communication, and 5) provider behaviour. Treating the patient as a whole and treating them with respect and empathy were key aspects of provider behaviour, which together with other facilitators, such as effective communication, worked to facilitate PCC. These findings are supported in a narrative review of patient provider communication. King & Hoppe [114. ] suggested six functions as the ‘best practice’ during patient-provider consultation: 1) establishing a relationship, 2) obtaining information, 3) sharing information, 4) decision making, 5) promoting disease and treatment behaviours, and 6) the ability to respond to emotions.

This review also confirms previous research on PCC barriers conducted in different settings. A scoping review of studies based in Australia, Canada, Netherlands, Norway, Sweden, and the United States involving migrants and refugee women found that the barriers to PCC were determined by patient health and language literacy levels, health provider behaviours and knowledge (i.e., lack of knowledge about culture and religion), and organizational practices (i.e., lack of language services) [115. ]. Another systematic review of 23 qualitative studies involving children and young people in mental health services found that a lack of specialist knowledge, poor communication, and scarce resources hinder PCC implementation [116. ]. In addition to those practices already mentioned, these can be overcome by equipping health providers with confidence and knowledge through explicit training in PCC [116. ] and by modelling PCC behaviours and in workplaces that explicitly espouse key PCC values through providing specialized training courses to improve for instance communication skills [50. , 90. , 95. ]. Salary incentives and promotion opportunities could also be offered to motivate health providers to enhance their qualifications and improve their abilities to provide patient-centered care [98. ]. Creating organizational programs that emphasize decentralization, in addition to providing access to information, support, resources, and opportunity would enable PCC [58. ]. In addition, integrating the concept of patient and family-centered care within the educational curriculum [54. , 64. ] and providing students with opportunities to acquire the necessary skills needed for clinical practice [61. ] would further enable the adoption of PCC. Notably, establishing patient-centered care relies on changes associated with both patients and physicians therefore there is a need to improve health literacy which would support PCC practice where patients’ behaviour would change from passive to more involved in decision making [52. , 67. ].

### PCC for the MENA region

This paper has used existing research to map out the dimensions of current practices in patient centred care in the MENA region. Most studies did not employ relevant PCC frameworks or engage with the broader PCC literature. There are several existing frameworks that describe the dimensions of PCC. For example, Scholl et al. [117. ] have proposed an integrative model that can be used in different healthcare settings and health care education to design a curriculum focusing on PCC. Mead and Bower [118. ] also proposed dimensions that determine the effectiveness of patient-provider interactions. However, none of these frameworks were developed with reference to the MENA region or have been explored in relation to the region. In order to consider the scope of research practice on PCC in the MENA region we compared our findings with the eight dimensions of PCC introduced by the Picker Institute and the Commonwealth Fund (see background section) [19. , 43. ]. According to our findings, there had been little focused research on the dimensions of coordination of care, emotional support, physical comfort, and continuity of care in the MENA region. These dimensions are significant indicators of patients’ perception of the quality of care and there is therefore a need to focus on these dimensions in future research.

As described above there are considerable local contextual factors that mean that the model of PCC operating in the MENA region may be different to that conceptualised elsewhere. We have highlighted the key factor of culture and, in particular, the impacts of the role of the family in health care, the impact of practitioner social standing, and gender as being key to understanding the practice of PCC in the region. These should all be key to a revised conceptual definition of PCC in the MENA region.

## Conclusion

This systematic review is the first review conducted in the MENA region. From this discussion we can conclude that the health care system in the MENA is influenced by culture that impacts on the interactions between providers and patients, places a greater emphasis on family rather than individual patient involvement in care and emphasizes collectivism over individualism. Future research is needed to explore this further and investigate the association of culture and patient willingness to participate in decision making and PCC.

Our review indicates that there is support for adopting PCC in the MENA region, but that the practice of PCC is limited. This was acknowledged in multiple studies [52. , 83. , 91. , 95. ] in different care settings. We therefore conclude that considerable effort is still needed to ensure that health care in the MENA region is patient centered. The transition to patient-centredness requires a focus on patients, healthcare providers and organisations. However, we have seen that within the broad context of PCC, the patient's preference to participate in care varies depending on their cultural background.

This review showed that there is lack of a common definition of PCC in this region. The literature also showed that definitions must be adaptive to the local MENA context and should incorporate an understanding of culture which incorporates a focus on family involvement, the impact of the health practitioner's social standing, and gender. This understanding of PCC in the MENA region, including this developing definitional work, must be discussed in further research.

## Data Availability

The datasets used and/or analysed during the current study are available from the corresponding author on reasonable request.
